# Risk factors for acute complicated appendicitis in children aged three years and younger

**DOI:** 10.1186/s12887-024-04959-w

**Published:** 2024-07-27

**Authors:** Jun-Jun Ju, Tao Zhang, Yuan Cheng, Yu-Liang Zhou, Shi-Qin Qi, Zhen-Qiang Zhang, Wei-Chen Shen, Zhu-Bin Pan

**Affiliations:** 1 Department of General Surgery, Anhui Provincial Children’s Hospital, Children’s Hospital of Anhui Medical University, No.39 Wangjiang East Road, Baohe District Hefei, Hefei, Anhui 230000 China; 2 The Fifth Clinical Medical College of Anhui Medical University, Hefei, Anhui 230000 China

**Keywords:** Complicated appendicitis, Risk factors, Young children

## Abstract

**Objective:**

The aim of this study is to identify risk factors associated with acute complicated appendicitis (CA) in children aged three years or younger, providing a theoretical foundation for the management and treatment of acute appendicitis (AA).

**Methods:**

A retrospective analysis was conducted on 135 pediatric patients with AA, admitted to the Department of General Surgery at Anhui Children’s Hospital between December 2020 and December 2023, who underwent successful surgical treatment. Based on the intraoperative and postoperative pathological findings, patients were categorized into two groups: complicated appendicitis (CA) (*n* = 97 cases) and uncomplicated appendicitis (UA) (*n* = 38 cases). Clinical data including gender, age, weight, disease duration, preoperative white blood cell count (WCC), neutrophil granulocyte (NEUT) count, C-reactive protein (CRP) levels, total bilirubin (TBil) levels, procalcitonin (PCT) levels, calprotectin (Cal) levels, preoperative ultrasound results indicating the presence or absence of fecaliths, maximum appendix diameter, and pediatric appendicitis sore (PAS) were collected and analyzed. Comparative analysis was performed to investigate the differences between the groups and identify risk factors of CA.

**Results:**

The CA group exhibited significantly higher values in disease duration, CRP levels, PCT, Cal, presence of appendiceal fecaliths, maximum appendix diameter, and PAS compared to the UA group (*P* < 0.05). Multivariate analysis identified CRP levels, maximum appendix diameter, and PAS as independent risk factors for CA. Specifically, differences in CRP level (OR = 1.045, 95% CI:1.024 ~ 1.067, *P* < 0.001), PAS (OR = 1.768, 95% CI:1.086 ~ 2.879, *P* = 0.022), and maximum appendix diameter (OR = 1.860, 95% CI:1.085 ~ 3.191, *P* = 0.024) were significant. The area under the receiver operating characteristic curve values were 0.6776 for the PAS, 0.7663 for CRP, and 0.5604 for the maximum appendix diameter.

**Conclusion:**

CRP levels, PAS, and maximum appendix diameter are independent risk factors for CA in children under three years of age. These parameters are valuable for the early diagnosis of CA.

## Introduction

Acute appendicitis (AA) is a prevalent abdominal surgical emergency in children, classified into complicated appendicitis (CA) and uncomplicated appendicitis (UA). CA is often associated with poor prognosis and may involve complications such as appendiceal gangrene, perforation, or periappendiceal abscess [1]. Although the overall incidence of AA in young children is relatively low, perforation rates are significantly higher in this age group (< 1 year old, 100%; 1–2 years old, 100%; 2–3 years old, 83.3%; 3–4 years old, 71.4%; 4–5 years old, 78.6% and 5 years old, 47.3%) [2]. Moreover, Nandan et al. [3]. reported that the risk of appendiceal perforation in young children increases with disease progression, underscoring the importance of early diagnosis of CA.

In recent years, numerous clinicians and researchers have endeavored to establish methods for assessing the severity of appendicitis. However, prior studies have demonstrated inconsistency in the early diagnosis of CA among young children. Laboratory investigations, including C-reactive protein (CRP), preoperative white blood cell count (WCC), total bilirubin (TBil), and calprotectin (Cal), have demonstrated significance in studies involving adult CA but exhibit limited specificity and sensitivity in young children [4]. Imaging tests and appendicitis scoring systems have also been employed to facilitate early diagnosis of CA, yet consensus remains elusive. Abdominal ultrasound lacks specificity, while computed tomography can expose young children to significant ionizing radiation. [5, 6] Dependence solely on the Pediatric Appendicitis Score (PAS) can lead to misdiagnosis or failure to diagnose, particularly in cases with atypical clinical presentations [7]. Consequently, early assessment of CA in young children remains a significant challenge.

Therefore, the purpose of this study is to investigate the risk factors associated with acute CA in children, providing a theoretical foundation for the early diagnosis, management, and treatment of CA.

## Data and methods

### General data

**Fig. 1:**
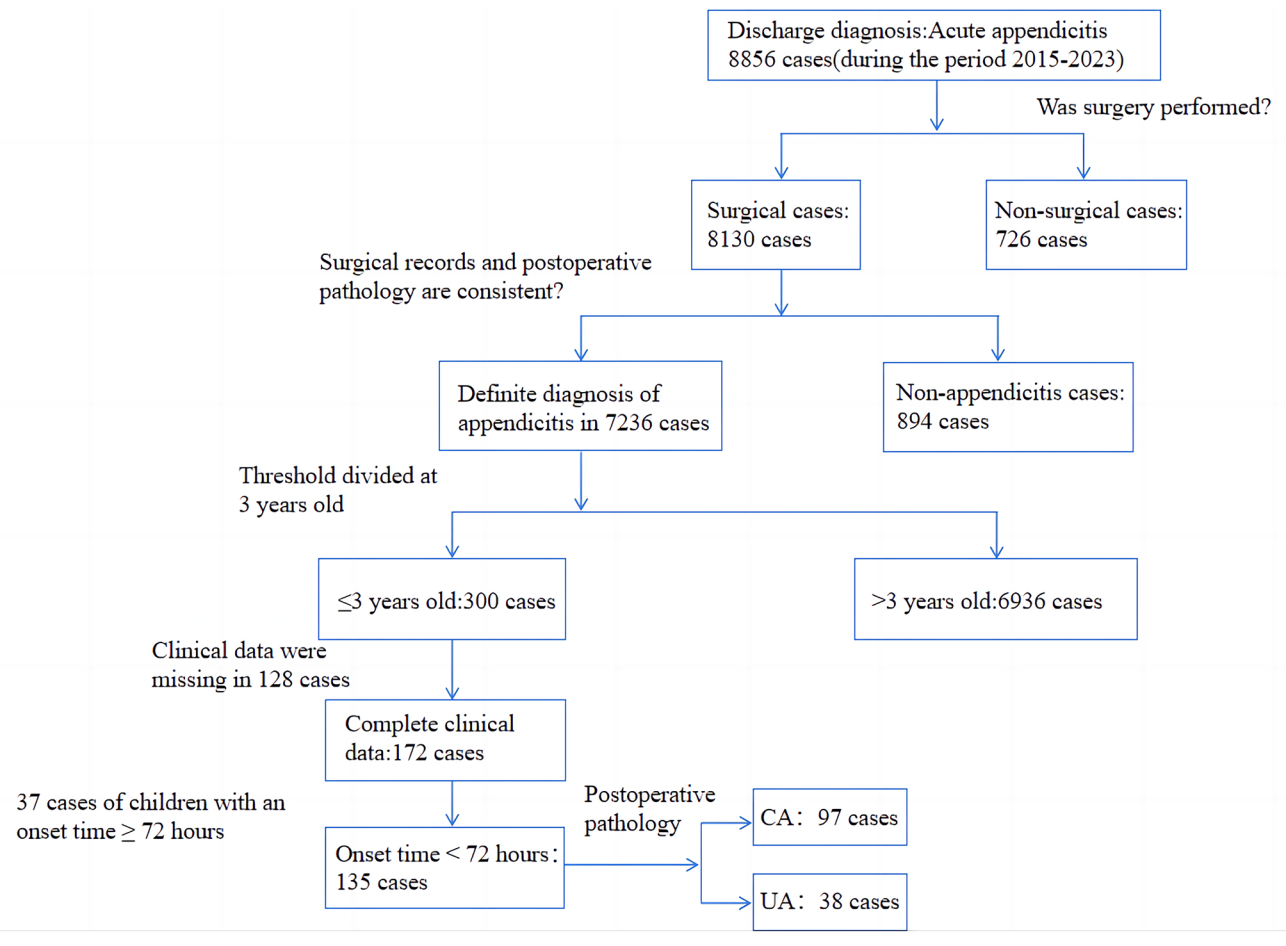
Study flow chart. Abbreviation: complicated appendicitis (CA), uncomplicated appendicitis (UA).

This retrospective study included 135 pediatric patients diagnosed with AA and surgically treated at the Department of General Surgery, Anhui Children’s Hospital, from December 2020 to December 2023. Based on intraoperative and postoperative pathological findings, the patients were classified into two groups: CA group (*n* = 97 cases) and UA group (*n* = 38 cases) (Fig. 1). The study was conducted in accordance with the principles of the Declaration of Helsinki, and the study protocol was approved by the Anhui Provincial Children’s Hospital’s ethics committee. The need for informed consent was waived by the ethics committee/Institutional Review Board of Anhui Provincial Children’s Hospital, because of the retrospective nature of the study. The requirement of patient consent for inclusion was waived. Patient personal privacy and data confidentiality has been upheld.

### Inclusion and exclusion criteria

Inclusion criteria: (1) Presence of either typical or atypical pain in the right lower abdomen, with or without rebound pain and muscle tension; (2) Elevated levels of WCC, CRP, and other inflammatory indicators, or ultrasound indicating appendix thickening and increased echogenicity of the surrounding fat; (3) Disease duration < 72 h; (4) Age ≤ 3 years; (5) AA diagnosis confirmed based on postoperative pathology; and (6) Complete clinical data.

Exclusion criteria: (1) Disease duration ≥ 72 h; (2) Presence of other inflammatory diseases, such as urinary system infection, respiratory system infection, etc.; (3) Presence of immunodeficiency diseases; and (4) Chronic appendicitis and acute exacerbation of chronic appendicitis.

Parameters and diagnostic criteria for CA and UA [8].

Criteria for UA: Intraoperative findings of UA included appendix thickening, mucosal redness, and edema. Postoperative pathological indication for UA encompassed simple appendicitis or suppurative appendicitis without perforation. Simple appendicitis was characterized by macroscopic hyperemia and edema of the appendix, with microscopic features showing inflammatory cell infiltration in the mucosa and submucosa. Acute suppurative appendicitis without perforation exhibited macroscopic evidence of appendix abscess formation, with microscopical findings revealing extensive neutrophil infiltration from the mucosal layer through to the muscle and serosal layers.

Criteria for CA: During surgical exploration for CA, findings revealed severe swelling, necrosis, perforation, and purulent discharge of the appendix. CA includes suppurative appendicitis with perforation, gangrenous appendicitis, and periappendiceal abscess. Pathologically, CA is characterized by macroscopic evidence of appendix wall perforation with abscess formation, localized gangrene, and circumappendiceal abscesses. Microscopically, extensive infiltration of inflammatory cells throughout all layers is observed, predominantly neutrophils but also including lymphocytes, plasma cells, and macrophages.

### Study indicators

During hospitalization, the following clinical data were collected and analyzed: gender, age, weight, disease duration, preoperative WCC, neutrophil granulocyte (NEUT), CRP, TBil, procalcitonin (PCT), Cal, preoperative ultrasound results indicating the presence or absence of fecaliths, maximum appendix diameter, PAS, follow-up time, and postoperative complications.

The disease duration refers to the period from the initial onset of clinical symptoms to the outpatient visit. The PAS was assessed by senior physicians and subsequently compiled, organized, summarized, and analyzed by the researchers involved in this study. The PAS consists of 8 variables with a cumulative score of 10 points. These variables include: cough/percussion/hopping tenderness in the right lower quadrant of the abdomen (2 points), anorexia (1 point), pyrexia (1 point), nausea/emesis (1 point), tenderness over the right iliac fossa (2 points), leukocytosis (1 point), polymorphonuclear neutrophilia (1 point), and migration of pain (1 point) [9]. 

### Antibiotic administration

All children received cephalosporin antibiotics (cefoxitin sodium or cefoperazone sulbactam sodium) once prior to surgery, administered at a dose of 40–160 mg/kg q8h. Infusions were completed within 30 to 60 min before surgery, with the treatment plans adjusted as necessary based on post-surgical abscess culture results.

### Discharge criteria

Discharge criteria included resolution of clinical symptoms such as abdominal pain, vomiting, fever, diarrhea, and tenesmus. Abdominal tenderness and masses were absent, and laboratory indices (WCC and CRP) had normalized. Imaging results indicated no significant masses or effusions in the abdominal cavity.

### Primary and secondary outcomes

The primary outcome of this study was to identify independent risk factors for CA in young children with AA. Secondary outcomes included evaluating the diagnostic efficacy of these independent risk factors, measured by metrics such as the area under the receiver operating characteristic (ROC) curve (AUC), optimal cutoff values, sensitivity, and specificity.

### Statistical methods

Statistical analysis was conducted using IBM SPSS Statistics 26.0 software. All data were assessed for normal distribution, variance homogeneity, and independence. The results are expressed as mean ± standard deviation (x ± s) for the measurement data that conformed to a normal distribution. A two-sample t-test was employed to compare the continuous variables between groups. The count data are presented as frequency and percentage (n (%)). To compare categorical data between groups, either the chi-squared test or Fisher’s exact test was applied. Non-parametric data were analyzed using the Mann–Whitney U test. All independent variables were analyzed using one-way logistic regression, and variables that demonstrated statistical significance were then included in multivariate logistic regression analyses. Multivariate regression analysis utilized the ENTER method. ROC curves of the participants were plotted, and the AUC was calculated. The optimal cut-off value was determined using Youden’s Index. A two-sided *P* < 0.05 was considered to indicate a statistically significant difference. The 95% confidence interval (CI) for the odds ratio (OR) was calculated with a significance level set at *P* < 0.05.

## Results

### Baseline clinical characteristics and postoperative complications in children undergoing appendectomy

No significant differences were observed between the two groups in terms of gender, age, weight, WCC, NEUT, or TBil (*P* > 0.05). However, there were significant differences in the duration of disease, CRP, PCT, Cal, appendix fecalith, maximum appendix diameter and PAS (*P* < 0.05, Table 1).

The median follow-up duration was 7 months (interquartile range: 3 to 11 months). Postoperative complications although generally mild, were observed in all patients. These included infections, hemorrhages, appendicitis recurrence, fecal fistulae, peripheral tissue injuries, intestinal adhesions, intestinal obstructions, intraperitoneal abscesses, and poor incision healing. All complications resolved following appropriate symptomatic management, and no severe adverse events were recorded.

### Results of one-way logistic regression analysis

All variables were included in the univariate analysis. The findings indicated statistically significant differences in disease duration, CRP, PCT, Cal, appendiceal fecaliths, maximum appendix diameter, and PAS (*P* < 0.05, Table 2).

### Results of multivariate logistic regression analysis

Multivariate logistic regression analysis incorporated all the significant variables identified in the univariate regression analysis as independent variables. These variables encompassed disease duration, CRP levels, PCT levels, Cal, presence of appendiceal fecaliths, maximum appendix diameter, and PAS. The dependent variable in this analysis was the AA grouping (assignment: UA = 0, CA = 1). The results indicated that the PAS (OR = 1.768, 95% CI: 1.086 ~ 2.879, *P* = 0.022), CRP (OR = 1.045, 95% CI: 1.024 ~ 1.067, *P* < 0.001), and maximum appendix diameter (OR = 1.860, 95% CI: 1.085 ~ 3.191, *P* = 0.024) were independent risk factors for CA (Table 3).

### ROC curve analysis for PAS, CRP, and maximum appendix diameter

**Fig. 2:**
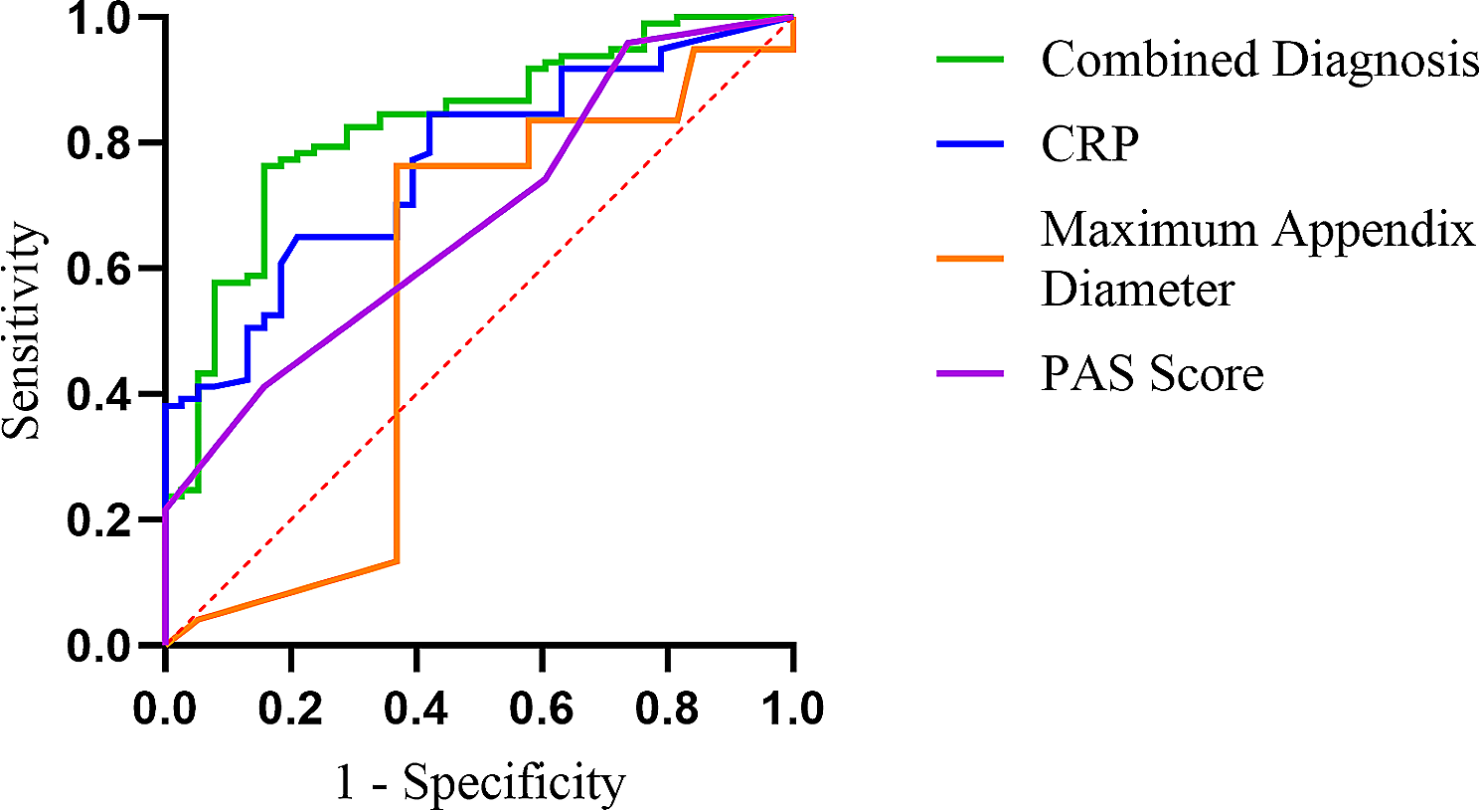
ROC curve of PAS, CRP, and maximum appendix diameter for CA diagnosis

In the ROC analysis for diagnosing CA, the AUC for the PAS was 0.678, with an optimal cutoff value of 7, sensitivity of 41.24% and specificity of 84.21%. For CRP, an optimal cutoff value of 50.22 mg/L was observed, with an AUC of 0.766, sensitivity of 64.95%, and specificity of 78.95%. The AUC for maximum appendix diameter was 0.560, with an optimal cutoff value of 7 mm, sensitivity of 76.29%, and specificity of 63.16% (*P* < 0.05). The combined prediction parameter had an AUC of 0.828, with a cutoff value of 0.73, (*P* < 0.05), demonstrating a combined prediction sensitivity of 76.29% and specificity of 84.21% (Fig. 2).

## Discussion

AA is a prevalent pediatric emergency, with an incidence of 2–9% in children under 6 years of age [5]. Specific incidence statistics for appendicitis in children under 3 years of age are not well-defined; however, it is generally accepted that early diagnosis is challenging due to atypical symptoms. Moreover, the disease progresses rapidly, leading to a notably high incidence of AA perforation in this age group [10]. Currently, the PAS is the predominant appendicitis scoring system used for young children [11]. However, several studies have indicated that the PAS fails to consider the disease duration and thus cannot be relied upon to accurately identify the types of AA in children younger than four years [12]. AA progresses rapidly in children, where even slight variations in its course can significantly impact prognosis and treatment.

Numerous physicians and researchers have made efforts in recent years to establish a method for determining the types of AA. Nandan et al. discovered that the risk of appendicitis perforation in young children increased as the duration of the disease progressed [3]. Küçükakçali et al. found that Cal may play an important role in the diagnosis of AA, with levels frequently elevated in children diagnosed with AA [13]. Sevgi et al. further proposed that serum Cal levels at a threshold of 670 ng/ml exhibit a sensitivity of 73.3% and specificity of 100% in distinguishing between simple and complicated AA [4]. Alfehaid et al. observed that elevated TBil and direct bilirubin were associated with CA, with TBil elevation demonstrating a sensitivity of 57.6% and specificity of 73.6% [14]. However, research regarding the method for determining the CA in young children remains limited.

Due to challenges associated with communicating and examining children under three years of age, clinicians frequently depend on imaging techniques and laboratory tests to supplement their assessments. Key diagnostic tools include leukocyte counts, neutrophil levels, PCT, CRP, and ultrasound. Based on the results of our study, we identified PAS, CRP, and maximum appendix diameter as independent risk factors in this context. The PAS is a comprehensive AA assessment tool that considers signs, symptoms, and hematologic tests, incorporates multiple factors, and demonstrates high accuracy. However, it has low sensitivity and specificity when used in isolation [7]. Despite its objective quantifiability, several researchers have continued to investigate its predictive value in CA. The PAS was able to distinguish the types of AA with an optimal cutoff value of 7 in this study, demonstrating a sensitivity of 41.24% and a specificity of 84.21%. Fujii et al. discovered that the PAS had a predictive cutoff value of > 8, which significantly improved the diagnosis of CA; [13] these results are consistent with those of the current study.

In our study, CRP emerged as an independent risk factor for CA in patients with AA. CRP is a group of acute-phase proteins that are produced in the body in response to inflammation. It is a sensitive indicator of the infection status and inflammatory response of the body. Numerous investigations have recognized its ability to differentiate the severity of inflammation. Van et al. discovered that CRP effectively distinguished between CA and UA, showing elevated levels correlating with disease severity, as indicated by an AUC of 0.72 [15]. The AUC of 0.766, sensitivity of 64.95%, and specificity of 78.95% for CRP in the present study were found to be similar to the values that had been previously reported. Afzal et al. retrospectively analyzed preoperative hematology and postoperative pathology in more than 2,000 cases of AA and found a strong correlation between CRP and the degree of inflammation, with high CRP levels predicting severe AA [16].

**Table 1:** Baseline clinical characteristics

Clinical characteristics	CA group (*n* = 97)	UA group (*n* = 38)	χ^2^/t	*p*
Males	55 (56.70%)	23 (60.53%)	0.16	0.686
Age (months)	27.17 ± 6.91	24.65 ± 7.48	-1.86	0.064
Weight (Kg)	11.13 ± 3.33	12.38 ± 3.61	1.92	0.057
Disease duration (d)	2.24 ± 0.43	2.06 ± 0.42	-2.30	0.023*
WCC (×10^9^/L)	13.69 ± 1.74	13.15 ± 1.22	-1.76	0.080
CRP (mg/L)	15.0 (8.00,50.17)	55.4 (49.11,69.33)	-4.806	< 0.01*
NEUT (%)	76.60 ± 11.53	72.86 ± 8.31	-1.56	0.076
TBil (umol/L)	9.96 ± 1.62	9.66 ± 1.74	-0.93	0.352
PCT (ng/L)	10.17 ± 9.65	5.67 ± 2.84	-4.16	< 0.01*
Cal (µg/g)	970.80 ± 160.50	899.90 ± 123.24	-2.45	0.015*
Appendiceal fecalith (cases)	44 (45.36%)	9 (23.68%)	-2.63	0.015*
Maximum appendix diameter (mm)	7.23 ± 0.91	6.87 ± 0.94	-2.05	0.043*
PAS (points)	6.39 ± 1.27	5.50 ± 1.06	-3.83	< 0.01*

*P < 0.05 indicates statistically significant difference. χ^2^ is the statistic value of the Chi-square test, and t is the statistic value of the T-test. Abbreviation: CA, complicated appendicitis; UA, uncomplicated appendicitis; WCC, white blood cell count; CRP, C-reactive protein; NEUT, Neutrophil granulocyte; TBil, Total bilirubin; PCT, Procalcitonin; Cal, Calprotectin; PAS, Pediatric appendicitis score

**Table 2:** Univariate logistic regression analysis of CA related risk factors

Clinical characteristics	CA group (*n* = 97)	UA group (*n* = 38)	B	OR (95% CI)	*p*
Males	55 (56.70%)	23 (60.53%)	-0.311	0.733 (0.398 ~ 1.584)	0.429
Age (months)	27.17 ± 6.91	24.65 ± 7.48	0.047	1.048 (0.996 ~ 1.103)	0.068
Weight (Kg)	11.13 ± 3.33	12.38 ± 3.61	-0.112	0.894 (0.796 ~ 1.005)	0.060
Disease duration (d)	2.24 ± 0.43	2.06 ± 0.42	0.979	2.663 (1.118 ~ 6.344)	0.027*
WCC (×10^9^/L)	13.69 ± 1.74	13.15 ± 1.22	0.233	1.262 (0.970 ~ 1.643)	0.083
CRP (mg/L)	54.99 ± 26.47	28.31 ± 22.62	0.040	1.040 (1.023 ~ 1.058)	< 0.001*
NEUT (%)	76.60 ± 11.53	72.86 ± 8.31	0.029	1.030 (0.992 ~ 1.069)	0.124
TBil (umol/L)	9.96 ± 1.62	9.66 ± 1.74	0.122	1.130 (0.874 ~ 1.460)	0.351
PCT (ng/L)	10.17 ± 9.65	5.67 ± 2.84	0.101	1.107 (1.023 ~ 1.198)	0.012*
Cal (µg/g)	970.80 ± 160.50	899.90 ± 123.24	0.003	1.003 (1.000 ~ 1.005)	0.019*
Appendiceal fecalith (cases)	44 (45.36%)	9 (23.68%)	-0.984	0.374 (0.160 ~ 0.873)	0.023*
Maximum appendix diameter (mm)	7.23 ± 0.91	6.87 ± 0.94	0.421	1.523 (1.009 ~ 2.299)	0.045*
PAS (points)	6.39 ± 1.27	5.50 ± 1.06	0.641	1.898 (1.324 ~ 2.722)	< 0.001*

*P < 0.05 indicates statistically significant difference. OR value and 95% CI greater than 1 indicate that this variable is a risk factor for CA. Abbreviation: B is the regression coefficient; OR, odds ratio; CI, confidence interval; CA, complicated appendicitis; UA, uncomplicated appendicitis; WCC, white blood cell count; CRP, C-reactive protein; NEUT, Neutrophil granulocyte; TBil, Total bilirubin; PCT, Procalcitonin; Cal, Calprotectin; PAS, Pediatric appendicitis score

**Table 3:** Multivariate Logistic regression analysis of CA related risk factors

Clinical characteristics	B	SE	Wald	OR (95% CI)	*p*
PAS (points)	0.570	0.249	5.249	1.768 (1.086 ~ 2.879)	0.022*
CRP	0.044	0.011	17.666	1.045 (1.024 ~ 1.067)	< 0.001*
Maximum Appendix diameter (mm)	0.621	0.275	5.085	1.860 (1.085 ~ 3.191)	0.024*

*P < 0.05 indicates statistically significant difference. OR value and 95% CI greater than 1 indicate that this variable is a risk factor for CA

Abbreviation: B is the regression coefficient; SE is the standard error; OR, odds ratio; CI, confidence interval; CA, complicated appendicitis; CRP, C-reactive protein; PAS, Pediatric appendicitis score

Appendix diameter is an important parameter on abdominal ultrasound, the preferred test for diagnosing AA. A widened appendix diameter often indicates inflammatory appendiceal swelling or fecalith impaction, which suggests a high likelihood of appendicitis. A diagnosis of appendicitis is generally suspected in children when the diameter of the appendix is greater than 6 mm on ultrasound. Treatment is then determined based on the physician’s clinical experience. However, the appendix in children under the age of 3 often exhibits a funnel-shaped morphology and infrequent obstruction, causing many risks factor analyses to omit appendix diameter as an indicator. Trout et al. reported that perforated appendicitis is more common in younger children [17]. One reason for this is that smaller appendix diameters can lead to early obstruction. Based on this, Tong et al. determined that a diameter greater than 10.1 mm, with an AUC of 0.63 (95% CI: 0.57–0.69), was the most important factor for differentiating between complicated and uncomplicated AA in children [18]. A potential drawback is that the results could be significantly biased by the quality of the examination conducted by the physician in the medical department. The use of other parameters, such as TBil, Cal, and appendiceal fecalith, is also controversial. There was no difference in TBil between the two groups in our study. A correlation was observed between Cal and appendiceal fecalith in one-way analysis (*P* = 0.019; *P* = 0.023); however, it did not reach statistical significance in multivariate logistic regression analysis. Several relevant studies have reported that elevated TBil, increased levels of Cal, and the presence of appendiceal fecaliths independently contribute to the risk of CA in patients with AA [19–21]. The selection of the these indices to differentiate between CA and UA in children should be carefully considered in this particular context.

In this study, our objective was to differentiate between CA and UA in young children (≤ 3 years old) by combining commonly used clinical scoring scales, laboratory examinations, and ultrasound assessments. Ultimately, we successfully identified the risk factors associated with CA. Nonetheless, it is imperative to acknowledge certain limitations in this study Firstly, the sample size was limited. Secondly, patients whose surgeries were unsuccessful were excluded, potentially introducing selection bias. Thirdly, certain observational measures, such as ultrasound and the PAS, are subjective, which may introduce information bias. Future research should focus on conducting multicenter, large-scale, prospective studies to validate the generalizability of these identified risk factors.

## Conclusion

In conclusion, PAS, CRP, and maximum appendix diameter are independent risk factors for CA in children aged three years and younger with AA. Assessment of these indicators can aid in evaluating the severity and type of AA, thereby guiding appropriate treatment decisions.

## Data Availability

The datasets generated and/or analysed during the current study are not publicly available but are available from the corresponding author (Zhu-Bin Pan) on reasonable request.

## Abbreviations

WCC: White blood cell count
NEUT: Neutrophil granulocyte
CRP: C-reactive protein
TBil: Total bilirubin
PCT: Procalcitonin
Cal: Calprotectin
PAS: Pediatric appendicitis score
AA: Acute appendicitis
CA: Complicated appendicitis
UA: Uncomplicated appendicitis
